# A theory of oral healthcare decision-making in Appalachia

**DOI:** 10.1371/journal.pone.0303831

**Published:** 2024-05-20

**Authors:** R. Constance Wiener, Christopher Waters, Ruchi Bhandari

**Affiliations:** 1 Department of Dental Public Health and Professional Practice, West Virginia University, Morgantown, West Virginia, United States of America; 2 Department of Dental Research, West Virginia University, Morgantown, West Virginia, United States of America; 3 Department of Epidemiology and Biostatistics, West Virginia University, Morgantown, West Virginia, United States of America; Hamadan University of Medical Sciences School of Dentistry, ISLAMIC REPUBLIC OF IRAN

## Abstract

**Introduction:**

People make oral healthcare decisions regardless of having partial information, misinformation, sources that deliberately mislead, or information that is culturally influenced. This is particularly true in the Appalachian culture where oral healthcare decision-making practices are not well understood by researchers and dental professionals. Despite efforts to improve dental care utilization, the Appalachia region remains low in oral healthcare utilization. There is a need for a theory to identify concepts in decision-making when seeking oral healthcare. The theory could be useful in creating oral health interventions. The study objective is to develop a theory to identify concepts that influence oral healthcare decision-making in Appalachia (OHDA).

**Methods:**

The researchers used a grounded theory qualitative study design to explain data for a theory of OHDA. Participants from Appalachia, in 20-minute interviews, provided insights into concepts that influence OHDA from August 22, 2017 to May 26, 2022. Notes/memos were written during and after the interviews and coding was conducted after the interviews. Open coding categories emerged through constant comparison of responses.

**Results:**

Five overarching concepts that embody OHDA were discovered: Affect (Level of Pain/Emotion/Stress involvement), Awareness, Trust/belief, Resources, and Risk Perception. All participants discussed the impact of social media toward these concepts.

**Conclusion:**

To influence a person’s OHDA, public health officials and researchers need to address the person’s affect, level of awareness, trust/belief, available resources, and risk perception. Social media is **very** important in awareness concerning oral health information. These factors are important to consider for similar research in oral healthcare utilization at the population level.

## Introduction

Healthcare decision-making theories have many cultural influences and metaphors. Historically, healthcare decision-making theories were influenced by traditions, accepted knowledge, religion, and the social worldview of communities/regions and the people trusted as knowledge keepers. Ancient Greeks represented healthcare as balancing blood, phlegm, yellow bile, and black bile—corresponding metaphorically to their worldview of balance in fire, air, water, and earth [1]. Therefore, healthcare decision-making was based upon balancing blood, phlegm, yellow, and black bile. In traditional Chinese culture, healthcare was metaphorically related to balancing flow (where the irrigation channels formed the metaphor) [1]. Therefore, healthcare decision-making was based upon balancing flow (Qi).

There are many recent decision-making theories. For example, the Rational Decision Theory proposes that there are unseen forces (metaphorically, the invisible hand), and decisions are made rationally for self-interest [2,3] using a right/wrong; yes/no algorithm. The Prospect Theory proposes that people use decision weights to maximize gains and minimize losses in decision-making [4]. A visualization framework decision-making theory uses a dual processor as a decision-making metaphor in which one processor involves automatic, easy decisions (Type 1 decisions), and the other processor involves contemplative decisions (Type 2 decisions) [5]. The Fuzzy-Trace Theory of Medical Decision-making proposes that although there are accurate, verbatim accounts that are available for sound healthcare decision-making, past fuzzy memories cue decision-making [6]. Artificial neural network analyses with agnostic theoretical frameworks are also commonly presented [7] and used.

With so many theoretical options, current researchers aim to use the best theories to explain scientific data. They collect high quality, scientific data to explain or theorize about health/disease while recognizing that cultural/personal perspectives can bias their research [8] and recommendations for healthcare decisions. The U.S. Centers for Disease Control and Prevention pledged to “base all public health decisions on the highest quality scientific data that is [sic] derived openly and objectively” [9]. Individuals are best served when healthcare decision-making is rooted in research data based upon a sound theoretical framework.

Having a sound-theoretical framework is often not the case [10]. When healthcare decision-making is based upon a few anecdotes, or a single study, or upon inaccurate beliefs, or rebased upon a social influencer’s/celebrity’s comments [11], people can be harmed. Professionals, teachers, parents, friends, books, journals, television, radio, and online sources may provide current evidence-based information; or they may be the source of out-of-date, inaccurate, or deceptive information. Additionally, there is skepticism about the motives of clinicians who may have a financial conflict of interest in providing particular healthcare information [12]. Poor health-related decision-making is a public health concern.

Currently, researchers theorize healthcare decision-making and action depends upon the specific disease/condition/activity [8]. For example, emotion (fear) may be the primary factor in cancer treatment decisions and following medical advice; whereas, stress reduction may be important in tobacco use rather than seeking help for tobacco cessation; and a positive affective state (pain/emotions/stress) may be important in pursuing physical activity routines. Adequate oral healthcare is necessary for wellness, although it is often neglected [13,14]. Helping people make good decisions concerning oral healthcare requires understanding how decisions originate. For example, what decisions led to the decline in past-year dental visits in the U.S.? [15]. There is also a need to determine how oral healthcare decision-making originates in specific cultural groups, like people of Appalachia, a region in which oral health is low and there is a history of limited preventive utilization [16] despite ongoing improvement efforts.

Historically, writers described people in Appalachia—an area with low population density and limited-service availability [17], as having common characteristics including living within a homogenous population under geographic conditions limiting social interactions to their locality. The terrain is rugged with poor roads, winter road closures, and significant distance to access areas outside of a locality, including accessing healthcare [18]. The culture embraced self-sufficiency, independence, and seeking medical help as a last resort [19]. Researchers described culturally ingrained, health-impairing customs such as reluctance/waiting to seek medical help rather than utilizing preventive services; and low priorities toward dentition [18]. In pre-21^st^ century Appalachia, the definition of health was equated with being active [20]. Therefore, being unable to work, or needing to use public funding was stigmatized [21]. For example, three-fourths of 1576 adults living in Appalachia rated themselves as healthy; however, objective measures of health indicated over two-thirds were: sedentary (65%), hypertensive (76%), overweight (73%), hyperlipidemic (79%), or had two disease conditions or poor health behaviors (57%-66%) [22].

With the mid-20^th^ century interstate road systems and television access, the region and its culture shifted. Although not all areas have internet, the 2021 infrastructure bill has federal monies for such projects. Appalachian residents have greater access to healthcare with better roads and expanding telehealth services. With these changes, there is a need for a theory to identify current concepts in oral healthcare decision-making in Appalachia (OHDA). Such a theoretical basis could be useful for oral health interventions. The objective of this study is to develop a theory to identify concepts that influence oral healthcare decision-making in Appalachia.

## Methods

A grounded theory qualitative study design with 20-minute participant interviews was initially approved by the West Virginia University Institutional Review Board (Protocol 1705602413) as an expedited protocol. The protocol status was reclassified to flex after the West Virginia University Institutional Review Board (Protocol 2112482426) determined that the initial submission now qualifies for approval under the WVU Flexibility review model. We used a composite of three grounded theory approaches (Glaserian, Straussian, and constructivist) [23] for the conceptual framework with two questions integral to the three approaches: “What is the main concern of participants in [oral healthcare decision-making]?” and “What would account for resolution?” [24]. We began with the Glaserian approach of data collection: open-ended sentences, memo-writing, no pre-set conceptual categories [25]. Open coding helped us fracture/sort the data. We continued with theoretical sampling to collect data until saturation (no new insights/data) so that a theory could reveal itself [26]. A structured framework emerged early in memo-writing and literature was accessed. These are characteristics of the Straussian approach. We then used the flexible, creative, adaptable constructivist approach of Charmaz in the construction of the theory [23].

### Participant recruitment

Participants were recruited based on theoretical sampling, and the sample size was determined by data saturation. Interested participants were provided a cover letter which explained the project and its purpose. Verbal explanations of the research followed, and consent was obtained verbally. Written consent was not obtained as the interview did not contain PHI, and the only link between the interviewee and the data provided would have been the participants’ name. The IRB approved of this method as having the greatest confidentiality of data security and having minimal risk to the participant.

Participants included licensed professionals (dental, dental hygiene, medical, pharmacy, law, health sciences, and psychology) undergraduate and graduate students, and lay people who were ages ≥18 years, residing in Appalachia and associated with WVU, JW Ruby Memorial Hospital/WVU Medicine and its associated Morgantown, West Virginia (WV) clinics. WVU is a hub for dental and healthcare in WV where people travel across the state for oral healthcare. The participants in this study (patients, students, and licensed professionals) represented a cross-section of Appalachia, its culture, and differences in dental knowledge and beliefs. No one was excluded by sex, gender, race/ethnicity, or religion.

We then followed the constant comparison method of grounded theory for rigor in which information became available in a sequence/chain, linked by referral from the one interviewee to the next. Chain referral sampling is a common qualitative research technique [27] and was effective for recruitment which started on August 22, 2017 and ended on May 26, 2022.

### Data collection, researchers and data analysis

The interviews were free-flowing oral health decision-making conversations that occurred in the interviewer’s office. Conversations were not recorded as Glaser suggested recoded interviews focus on the recorded data and not the researchers’ observations nor the interviewer/participant interaction [28]. The intended focus is the accuracy of the discovered truth and not exact spoken words [28]. No semi-structured interview guide was used, although prompting/guiding/triggering questions started interviews and encouraged participants to talk freely (“to spill”). Examples include: “Could you please tell me how you think people learn or are influenced about oral health choices?”, “Could you tell me your thoughts about how people learn about new things in oral health like implants, tooth whitening, cone-beam x-rays, invisible orthodontics, same day ceramic crowns, or recommendations such as that children under six months should not be given fruit juice?”

As the sampling progressed and categories emerged, initial questions were modified. Some examples are: “What are some influences that have been good for improving oral care decisions and use of information and what are some that are not so good?”, “What does it mean for a dentist to stay “current” in oral health care?”, “Is there anything else that may influence getting information about oral health, making decisions, and using information about oral health?”. New informants were included as new data occurred and the direction of the interviews followed the informants’ lead for deeper understanding. Interviews continued until saturation and data was recorded by hand in a bound laboratory notebook that was secured in a locked office with only the researchers having access for confidentiality.

Glaser acknowledged that analysts are human and can impact research with unintentional biases or interpretations [25,29]. Glaser argued that bias can be reduced if researchers carefully undertake coding. In qualitative research, researchers become instruments of data interpretation [30]. Therefore, researchers should be described as any other instrument.

In this study, the interviewer (RCW) embodied influences from her university’s ideals (service, curiosity, respect, accountability, and appreciation); social position within the university; female gender; non-Hispanic white race/ethnicity; a mature adult status; a high academic level (DMD, PhD); a background in research involving disparities; and birth and rearing in Appalachia. The voice from which the results were interpreted was through all authors. In addition to the interviewer, it included a male author with the same university ideals, white race/ethnicity; mature adult status; a high academic level (MS); a background in research involving disparities; and birth and rearing in Appalachia; and a female author with the same university ideals, Asian race/ethnicity; mature adult status, a high academic level (PhD, MPA), a background in research and living over two decades in Appalachia.

The close association of the researchers with the characteristics of the Appalachian population required special care in coding to avoid bias; however, it also helped in providing insights. Continual review of memos was important in remaining disinterested and avoiding imposing values. Coding resulted after writing and re-writing notes and memos, reading and re-reading comments, notes, and memos, and sorting and re-sorting data features.

Data were coded in an iterative, constant comparison process so that the codes were grounded in the participants’ words. Notes of what the participants said, and impressions were read line-by-line as data fragments; then coded for meaning. After the researchers met and discussed the data, a conceptual model “emerged” as five categories became evident with the sorting and review of the data

Data saturation was reached with fifteen participants. Repetition of comments, ideas, and narratives were noticed early in the interviews. By the tenth interview, no new data were being reported. The participants included twelve females (80%).

## Results and discussion

**Fig 1:**
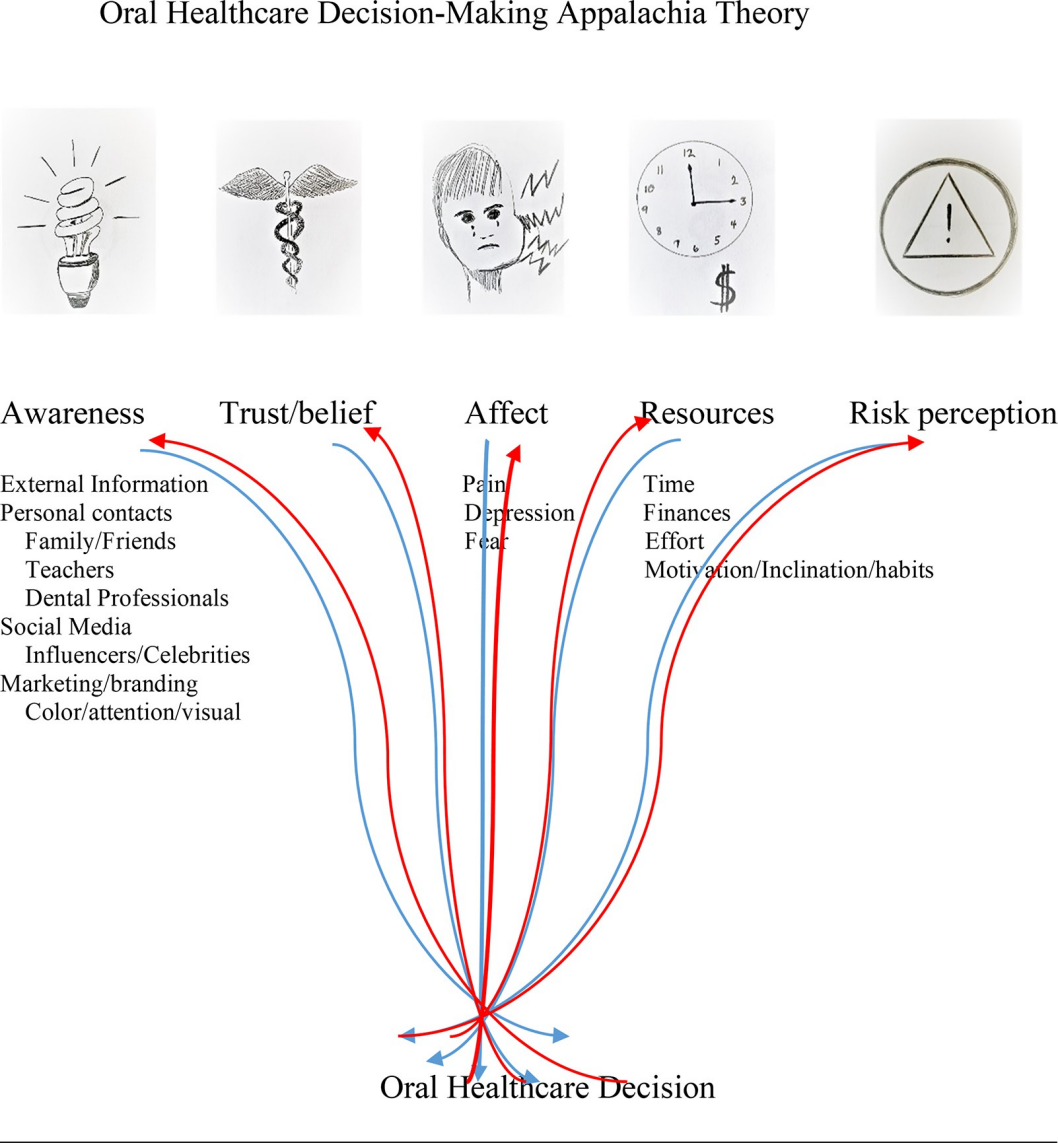
Categories of oral healthcare decision-making in Appalachia.

In OHDA, five categories became manifest from the latent data pattern (Fig 1):

- *Awareness*
- *Trust/belief*
- *Affect (Level of Pain/Emotion/Stress involvement)*
- *Resources*
- *Risk Perception*

### Core category

The main category expressed by participants, and in which participants responded with immediacy and a perceived sense of certainty and agency, was that awareness was brought by social media. The participants appeared excited when they discussed awareness. Participants reported that **awareness** of oral health issues was from many external sources (family, friends, teachers, and dental professionals) and social media influencers/celebrities were strong influences, especially for white, straight teeth. Participants noted that commercial advertisements, marketing, and branding of products for oral health (whiteners, whitening toothpaste, mouth rinses, teeth aligners, etc.) appeared throughout online sites, on television, and at sporting events. They reported commercial messaging was targeted, and had attention-grabbing graphics, vibrant colors, and celebrities/social influencers rather than through printed materials that required reading.

Some comments illustrating awareness were:

- *People learn about oral health from something that “catches their eyes; has pop-*
- *outs of color*, *or something that they happened to see;”*
- *“Influences come from Facebook…Some dentists don’t stay*
- *current;”*
- *“It needs to be exciting*. *They post it on social media;”*
- *“Pamphlets require too much effort…Brains shut off;”*
- *“…there is too much information to go through;”*
- *“There are brands everywhere;”*
- *“Everyone has a phone for information*.*”*

Participants reported they filtered information through **trust/belief.** If they did not trust the source, it was dismissed. Participants reported that trust/belief was important in formulating oral health decisions in the digital age.

Some comments illustrating the concept of trust/belief were:

- *“It is lucrative for the influencer;”*
- *“It’s like chain letters*. *Don’t break the chain;”*

The concept of the **level of affect** (pain/emotions/stress) emerged as an important factor. They reported intense, persistent oral pain motivated OHDA; while, fear, depression, and stress impeded it.

Some comments illustrating this concept were:

- *“It is hopeless*, *what is the use;”*
- *“Not going to the dentist—no need*, *no pain;”*
- *“Depression plays into motivation*.*”*

The concept of **available resources** was discussed in detail. Cost was important in OHDA. They talked about over-the-counter products that “if it didn’t work, it didn’t cost that much anyway.” Time as a resource was discussed. Participants indicated that OHDA was positive if there was little time that needed to be invested. They reported that “everyone” already knew about brushing and flossing, but time was the concern. Effort was closely related to time as a resource. Self-care behaviors requiring time and effort, such as flossing, were less likely to be decided upon, especially if people were tired/stressed. Dietary effort in avoiding sugary foods and beverages were reported as being influenced by affective state, financial resource, and effort. Additionally, habituation was an influence. While *previous success* with brushing/flossing, healthful diet, and dental visits was a positive resource, *habits* related to tobacco use, e-cigarette use, a diet high in sugary foods and beverages, and low use of dental services were indicative of having limited resources.

Examples of comments illustrating the concept of available resources were:

- *“Prices*!*;”*
- *“Cost;”*
- *“Self-motivation and time;”*
- *“Junk food costs less than regular food;”*
- *“Easy;”*
- *“Cheap is better—quick and cheap;”*
- *“Start young*. *Maintenance and it doesn’t take long*.*”*

The final concept, **risk perception**, was included due to the emergence from several comments relating to oral health such as:

- *“I know it is a risk*, *but I am taking it;”*
- *“People don’t see the risk;”*
- *“It may take a scare for someone*.*”*

Based on the above analyses of participant interviews, we surmise that awareness, trust/belief, affect, resources, and risk perception have independent and interactive effects in OHDA. The features of the OHDA are presented in the graphical abstract in which OHDA is presented as a complex, non-linear process involving awareness, trust/belief, affect, resources, and risk perception being perceived and acted upon through an individual filter.

The authors identified five concepts: Awareness, Trust/belief, Affect (Level of Pain/Emotion/Stress involvement), Resources, and Risk Perception. The associated metaphor is a worldview of interconnectedness and filters. Although a similar oral healthcare decision-making theory was not identified in the literature, these findings share concepts with research conducted in other health decision-making research. In chronic kidney disease decision-making theory, concepts were awareness, communication, culture/language/religion, and family [31]; in cancer decision-making, the concepts are empowerment through information-seeking, coming to terms, and choosing options [32].

Participants indicated that the first information received (primacy) held much importance as it “anchored” other information to it for comparisons [5]. Marketing strategists use anchoring (providing a manufacturers’ suggested retail price) to influence decision-making toward purchases. Participants in this study indicated social influencers/celebrities introducing items influenced OHDA with their early product introductions.

Trust/belief was important. Other researchers also identified trust as significant [33]. In oral healthcare, lower levels of trust increased dental avoidance and poorer oral health [34]. Lack of trust was an important concern during the COVID-19 pandemic. In one qualitative study, patients had less trust as exemplified by their questions on infection control practices [35]. Less trust was associated with unnecessary dental avoidance [36].

Affect was a self-evident concept. Pain is a primary reason to seek help. The oral health decision to seek care occurs when acute pain supersedes other factors and cannot be ignored. However, other affective states (fear, depression, anxiety, stress, etc.) have impacts on avoidance [37–39]. Oral healthcare avoidance has been particularly high during the COVID-19 pandemic due to anxiety [36] and fear [40].

Resources (financial, time, motivation) are important in oral health decision-making. A systematic review identified resource constraints as common reasons for public health decision-making [41]. One identified resource was motivation/habits. Habits (helpful or harmful) are difficult to break [42]. A consumer psychology research review examined forces interfering with healthful eating and highlighted the role of habits in both healthful and unhealthful diets [42].

Online, consumer-based websites are often ranked above informational websites allowing individuals to be influenced by their digital environment to make spurious decisions about healthcare options without input from healthcare providers [43]. Findings from our study illustrate the importance of risk perception when people make oral health decisions.

The health decision-making process consists of multiple, interlinked categories. Risk perception is critical. OHDA involves the individual’s perception of risks and benefits based upon the individual’s lived experiences, science, and research. Risk analysis has had a long history of inclusion in decision-making situations as different and far ranging as economics, gambling, farming, life choices, activities, and healthcare. In oral healthcare, risky behaviors include tobacco use, alcohol use, excessive sugar consumption, lack of oral hygiene, and lack of preventive care, among others. These behaviors generally do not have an immediate effect and many people do not recognize their long-term consequences.

Participants shared the importance of how information is presented. They agreed that eye-catching graphics rather than text was more influential. This observation was also reported in a review that attempted to communicate complex medical information visually [5]. In a study on the effect of graphic warnings on the purchase of sugary drinks, researchers discovered that compared to textual information, pictorial information had a stronger effect in reducing purchases [44]. Compared to cognitive strategies, attractive displays and vivid descriptions are more effective in reducing unhealthful consumption [45]. For these reasons, the theoretical model is displayed in the graphical abstract.

### Limitations

Although a majority of participants were female, the purpose of using grounded theory is to exhaust new ideas and reach saturation. We had a cross-section of Appalachia, its culture, and differences in dental knowledge and beliefs based upon a sequence/chain linked referral of one interviewee to the next. No one was excluded by sex, gender, race/ethnicity, or religion. New information was generated irrespective of participant sex. Saturation was achieved with data redundancy after the tenth interview and an additional step that could have strengthened this study would have been to formally conduct interviews 11–15 as post-saturation interviews.

### Implications

The findings of this study provide an important theory of OHDA

that could help develop oral health interventions. Our theory implies it is important to consider a broad, complex metaphor impacting OHDA.

## Conclusion

Empirical data were examined qualitatively using grounded theory to determine a theory of OHDA. Five concepts emerged for OHDA: awareness, trust/belief, affect, resources, and risk perception. Social media and graphical/visual presentations were identified as important in addressing awareness about OHDA.
